# Diagnostic performance of confocal laser endomicroscopy for optical diagnosis of gastric intestinal metaplasia: a meta-analysis

**DOI:** 10.1186/s12876-016-0515-3

**Published:** 2016-09-05

**Authors:** Xing-kang He, Dan Liu, Lei-min Sun

**Affiliations:** 1 Department of Gastroenterology, Sir Run Run Shaw Hospital, Zhejiang University Medical School, Hangzhou, 310016 China; 2 Institute of Gastroenterology, Zhejiang University (IGZJU), Hangzhou, 310016 China; 3 Department of Statistics, Texas A & M University, College Station, TX 77843 USA

**Keywords:** Confocal laser endomicroscopy, Intestinal metaplasia, Diagnostic performance

## Abstract

**Background:**

Gastric intestinal metaplasia (IM) is generally considered as a precancerous condition, a related risk factor for intestinal-type gastric cancer. However, an accurate endoscopic diagnosis of IM is a clinical challenge. Confocal Laser Endomicroscopy (CLE) is a newly technique that can provide real-time magnified images and visualize tissues at cellular or subcellular levels. The aim of this study is to clarify the diagnostic value of CLE in detection of IM in patients at high risk of gastric cancer.

**Methods:**

Systematic literature searches up to April 2015 in PubMed, Embase, Web of Science, Cochrane Library databases were conducted by two reviewers independently. The Quality Assessment of Diagnostic Accuracy Studies-2 (QUADAS-2) tool was applied to assess study quality and to reduce potential bias. A meta-analysis using Meta-Disc (version 1.4) and STATA software (version 13) was performed.

**Results:**

A total of four studies enrolled 218 patients and 579 lesions were included in this meta-analysis. On per-lesion basis, the pooled sensitivity and specificity of CLE were 0.97(95 % confidence interval (CI) = 0.94–0.98) and 0.94 (95 % CI = 0.91–0.97) respectively. The pooled positive likelihood ratio (PLR) and negative likelihood ratio (NLR) were 15.20 (95 % CI = 9.46–24.41) and 0.04 (95 % CI = 0.02–0.07) respectively. The pooled diagnostic odds ratio (DOR) was 479.59 (95 % CI = 205.64–1118.51) and summary receiver operating curve (SROC) area under the curve was 0.9884. There was no statistical significance of publication bias.

**Conclusion:**

CLE is a promising endoscopic tool in the detection of IM with the relatively high diagnostic value in patients at high risk of gastric cancer.

## Background

Gastric cancer is an aggressive disease, which is the second leading cause of cancer-related death globally [1]. It is well acknowledged that the pathogenesis of stomach cancer is a multi-step and sequential process beginning with chronic atrophic gastritis, going through intestinal metaplasia (IM), intraepithelial neoplasia and finally developing into adenocarcinoma [2–5]. The IM is generally considered as a premalignant lesion contributing to the development of gastric tumor [6, 7]. Current diagnosis of IM is based on pathological assessment of biopsy specimens with white-light endoscopy [8]. This conventional method is time-consuming and inefficient. It fails to detect IM whose mucosal surface looks normal. Improved endoscopic techniques such as chromoendoscopy, magnifying endoscopy, narrow-band imaging (NBI) technique have been shown to improve detection and diagnosis of IM during endoscopy [9, 10]. Above all techniques, however, are suboptimal for the detection of IM. None of them can distinguish the structure of individual cells or microstructures, and so pathologic assessment is still required [11, 12]. Recently, a new endoscopic technique called Confocal Laser Endomicroscopy (CLE) is applied to the detection of many gastrointestinal diseases [13–16]. It combines conventional white-light endoscopy with confocal laser microscopy and can be divided into two types:endoscope-based CLE (e-CLE) and probe-based CLE (p-CLE) [17]. The greatest advantage of CLE is to simultaneously provide macroscopic and microscopic images of the gastrointestinal epithelium [18]. The CLE has been reported to reveal high diagnostic value for digestive diseases [13, 14, 19, 20]. However, a comprehensive systematic review of the diagnostic performance of CLE on IM has not been reported.

In this meta-analysis, the goal is to establish diagnostic accuracy of CLE in diagnosis of IM in the high-risk group of gastric cancer.

## Methods

### Search strategy

A systematical search was conducted on PubMed, Web of Science, Embase, Cochrane Library databases to collect relevant articles published before April 2015. The search terms were: (“confocal endoscopy” OR “confocal laser endomicroscopy” OR “CLE”) AND (“IM” OR “intestinal metaplasia”). To avoid missing studies, we also examined the reference lists of all related articles for any additional papers. Two authors extracted the data from these articles independently. Authors of these articles were contacted by email if further detailed information are needed.

### Selection criteria

Articles were included in studies if they met all the following criteria: (1) using CLE to evaluate diagnostic accuracy of IM; (2) containing available data for constructing contingency tables for true positive (TP), false positive (FP), false negative (FN) and true negative (TN); (3) applying histopathology as a reference standard. Articles that met any one of the following criteria were excluded: (1) Insufficient data to construct contingency tables; (2) No histological diagnosis of lesions; (3) Reviews, case reports, abstracts and editorials.

### Data extraction and quality assessment

TP, FP, FN and TN from original studies were independently extracted to construct 2 × 2 tables by two reviewers. First author, publication year, country, number of patients, number of lesions, patients’ ages, sex ratio, histological diagnosis, number of endoscopists and endoscopes used from each studies were also extracted from studies. Discrepancies were resolved by discussions. The Quality Assessment of Diagnostic Accuracy Studies-2 (QUADAS-2) [21] was applied to assess study quality and potential bias. The QUADAS-2 tool contains four key domains that are rated in terms of the risk of bias. Quality assessment of the included studies was performed by two reviewers independently.

### Statistical methods

Data were analyzed by Meta-Disc (version 1.4) and STATA software (version 13). The pooled sensitivity, specificity, PLR, NLR and DOR were estimated by a fixed-effect model (Mantel–Haenszel method). Heterogeneity across the studies was tested by the Cochrane Q test. Inconsistency (I^2^) was used to express the percentage variability attributable to heterogeneity. I^2^larger than 50 % indicates heterogeneity is statistically significant, and P-values less than 0.1 illustrates the presence of heterogeneity among studies. The SROC and the area under the curve (AUC) were also constructed to illustrate the diagnostic precision of CLE. Finally, a funnel plot was constructed by Deeks’ asymmetry test to evaluate publication bias of selected studies.

## Results

### Included studies

**Fig. 1:**
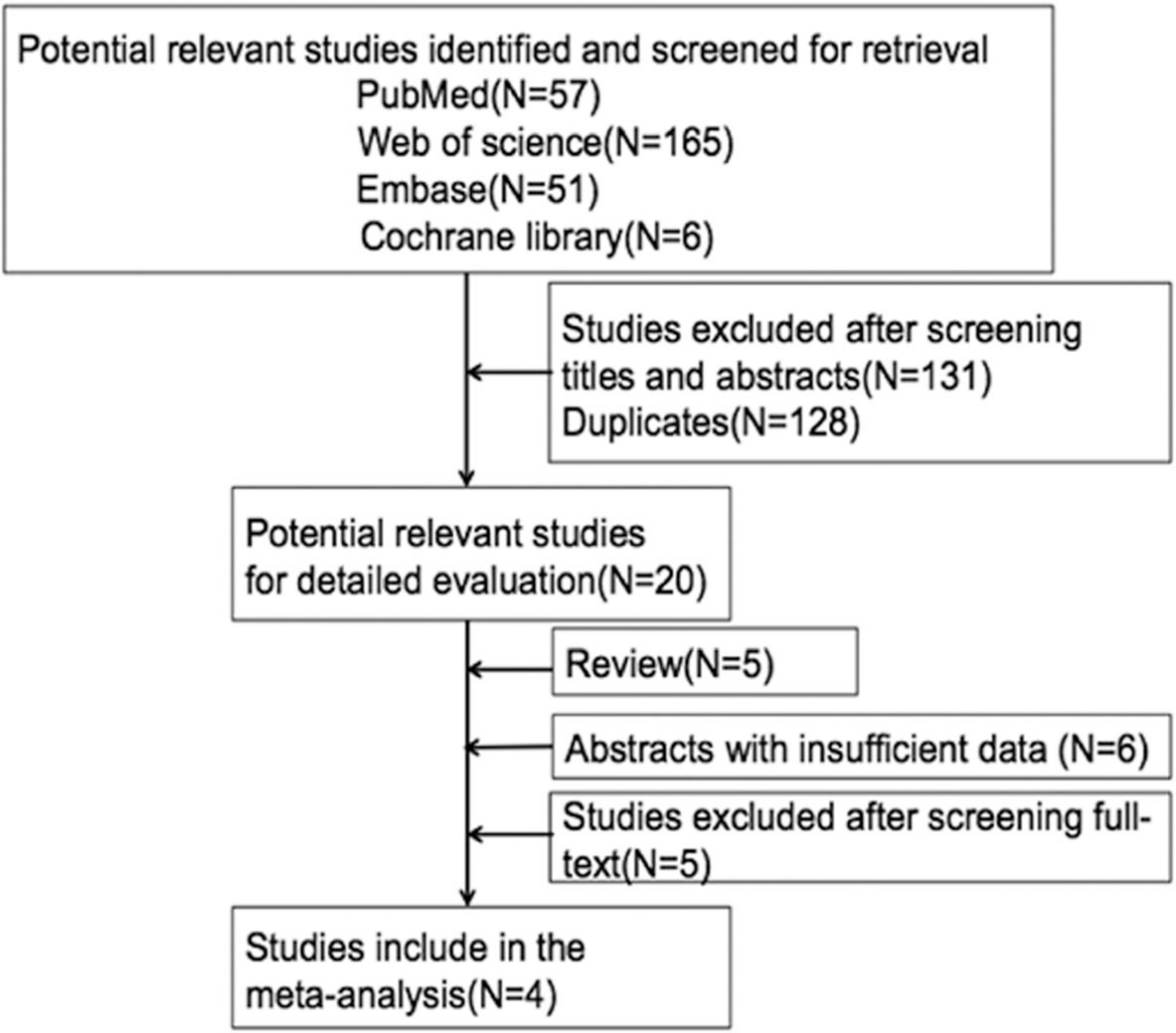
Literature search flow diagram

Four eligible studies were selected from the literature after searching the databases [22–25]. Fig. 1 shows how studies were screened from the literature. Overall 218 patients were enrolled in final analysis, with average of 54.5 patients per study (range from 20 to 85 patients). Information of per-lesion but not per-patient analyses was provided by all included studies. The CLE and white-light endoscopy were used to diagnose IM in all of included studies plus virtual chromoendoscopy was also performed in one study [24]. Additionally, virtual chromoendoscopy magnifying flexible spectral imaging color enhancement (ME-FICE) and p-CLE were applied to detect GIM in another study [20]. The main findings of the studies were presented in Table 1.

**Table 1:** Characteristics of the selected studies

Study(Year)	Country	Numbers of patients,n	Lesions examined, n	Mean age,year	M/F*	Histological reference standard	Endoscopists Number,n	Type of CLE system
Guo etal [27] (2008)	China	53	267	51	38/15	IM**	3	e-CLE
Zhen etal [29] (2013)	China	85	67	55	45/40	IM	3	e-CLE
Rapat etal[20] (2013)	Thai land	60	120	62.8	33/27	IM	5	P-CLE
Lim etal [19] (2013)	Singapore	20	125	62.5	15/5	IM	1	P-CLE

*IM* intestinal metaplasia *M/F* male to female, *CLE* Confocal Laser Endomicroscopy, *e-CLE* endoscope-based, *CLE P-CLE* probe-based CLE

### Study characteristics and quality assessment

According to the QUADAS-2 criteria, the quality of the inclueded studies was shown in Table 2. Generally, most of included studies met the quality criteria.

**Table 2:** Quality of articles using the QUADAS tool

Study	Risk of bias			Applicability Concerns			
	Patient selection	Index test	Reference standard	Flow and timing	Patient selection	Index test	Reference standard
Guo etal [27] **(**2008**)**	L	L	L	L	L	L	L
Zhen etal [29] **(**2014**)**	L	L	L	L	L	L	L
Rapat etal [20] **(**2013**)**	H	L	L	L	H	L	L
Lim etal [19] **(**2013**)**	L	L	L	L	L	L	L

*L* low risk; *H* high risk; *U* nuclear risk

### Diagnostic performance of CLE

**Fig. 2:**
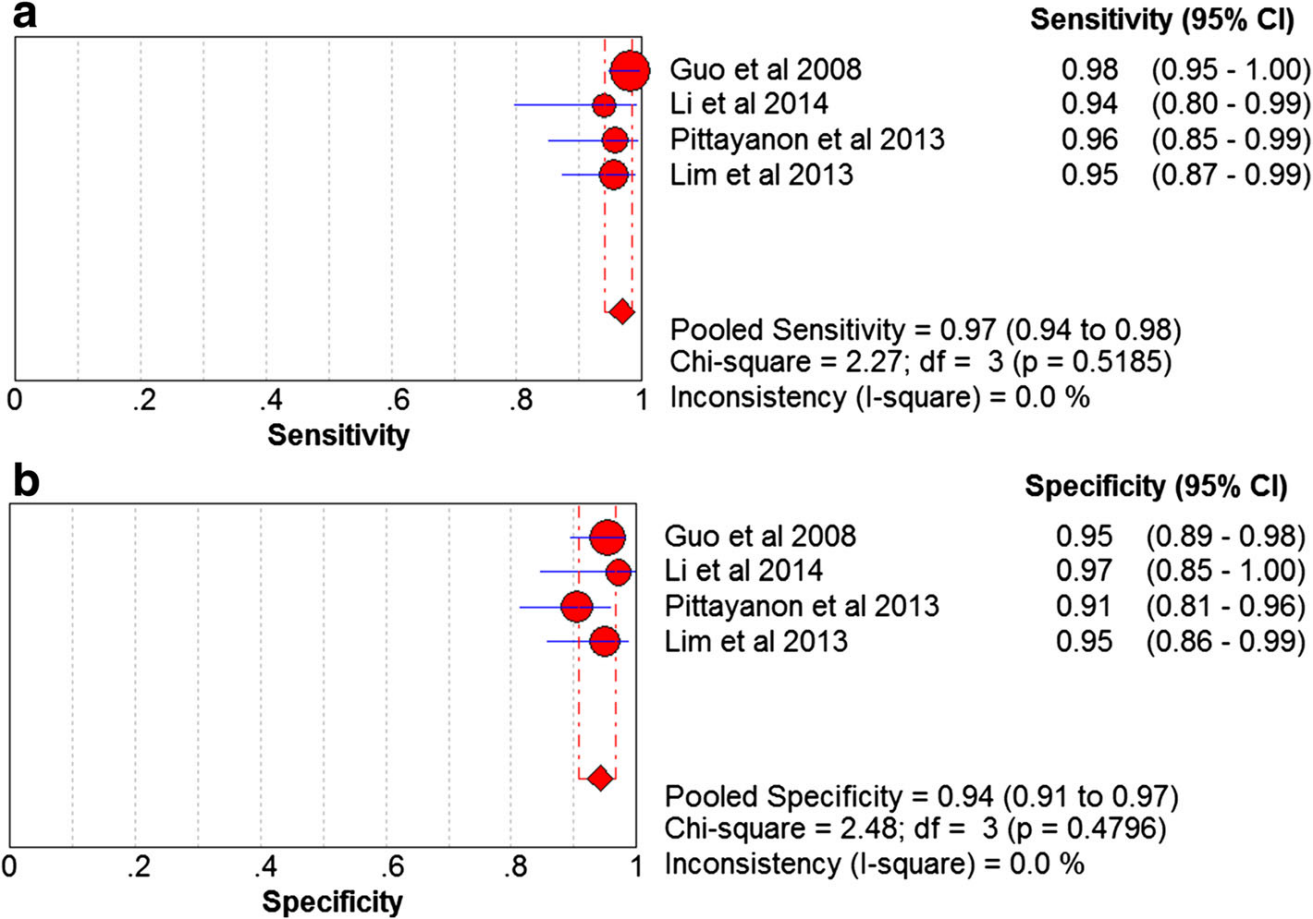
Forest plot showing pooled sensitivity and specificity for CLE to diagnose IM. **a** forest plots of the sensitivity; **b** forest plots of the specificity

**Fig. 3:**
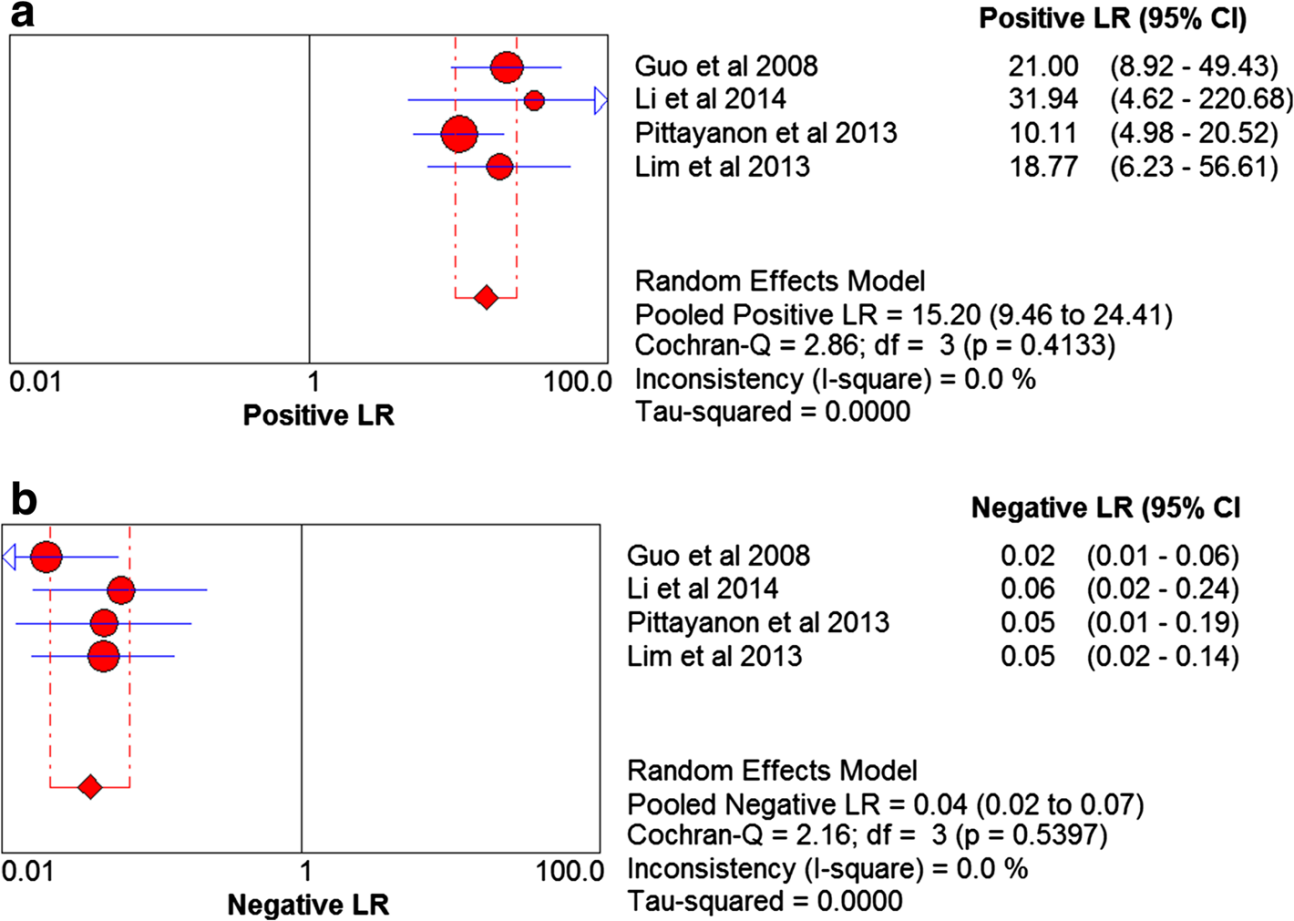
Forest plot showing positive LR and negative LR of CLE for IM. **a** forest plots of the positive LR; **b** forest plots of the negative LR. LR, likelihood ratio

**Fig. 4:**
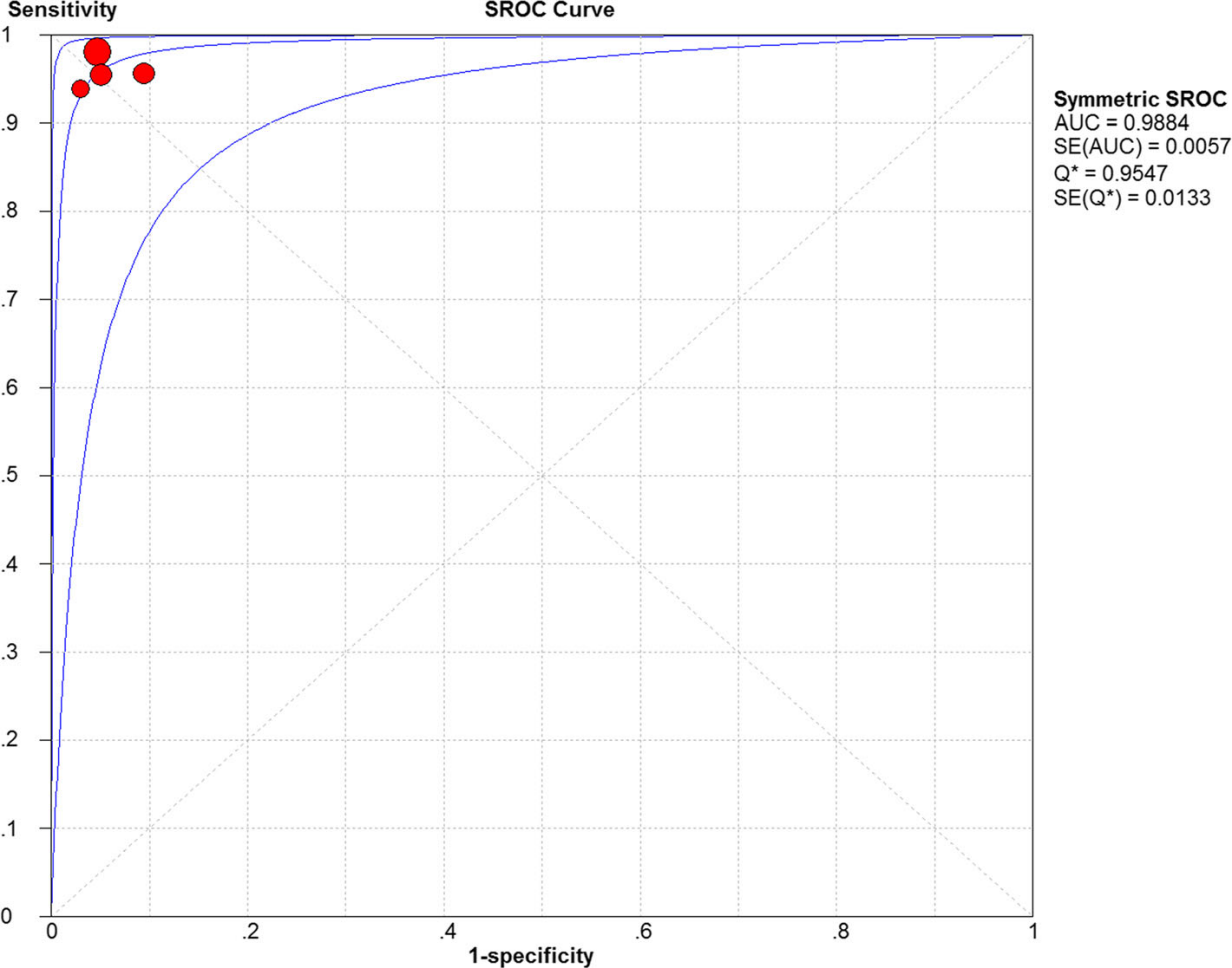
SROC curve of CLE for IM. SROC, summary receiver operating characteristic, AUC: Area under curve; SE: Standard error

**Fig. 5:**
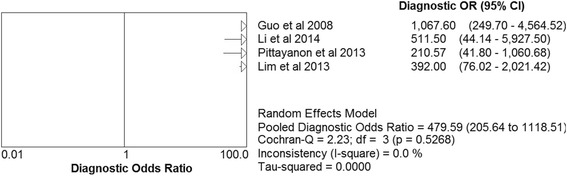
Forest diagnostic odds ratio of CLE for IM

**Fig. 6:**
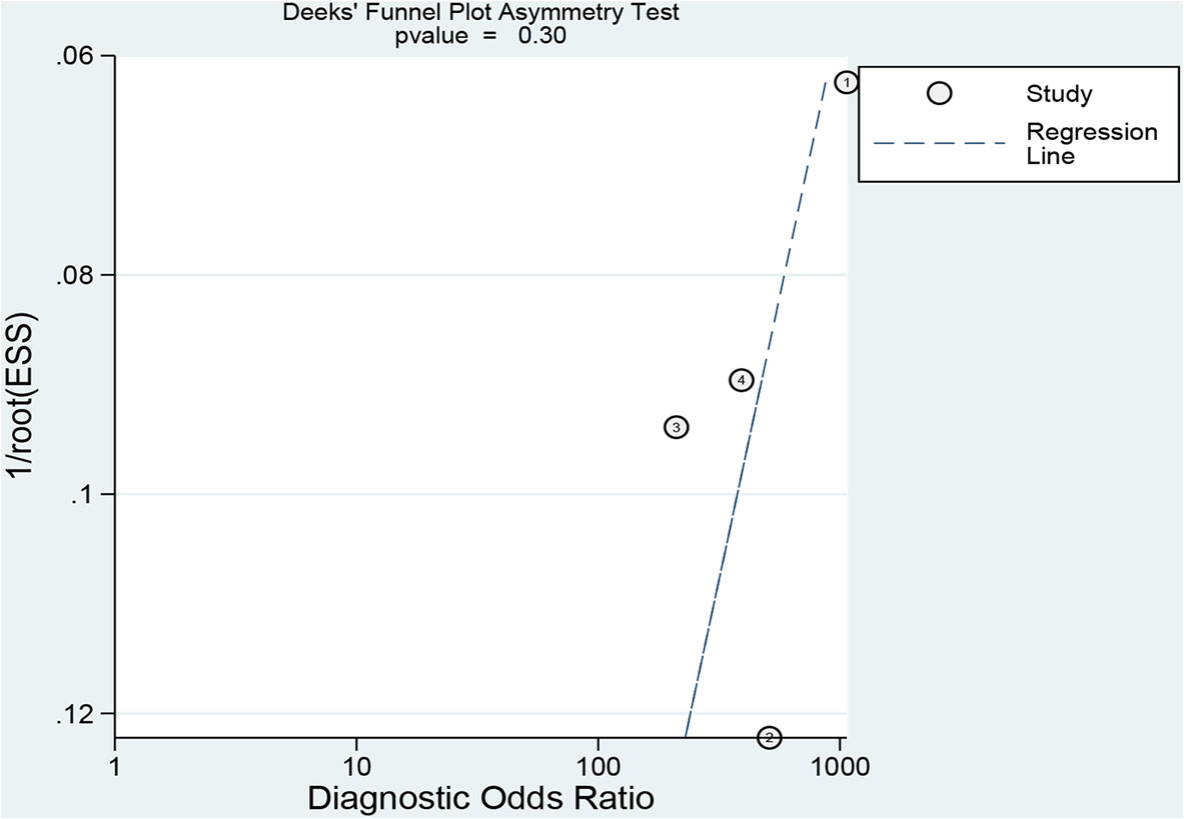
Deeks’ funnel plot to assess publication bias of selected studies

Based on the data from the four studies enrolling 218 participants and 579 lesions, the pooled sensitivity and specificity of CLE on per-lesion level were 0.97 (95 % CI =0.94–0.98) and 0.94 (95 % CI = 0.91–0.97) respectively (Figs. 2a and b). The pooled PLR was 15.20 (95 % CI = 9.46–24.41), and the pooled NLR was 0.04 (95 % CI = 0.02–0.07) (Figs. 3a and b). The AUC and pooled DOR were 0.9884 and 479.59 (95 % CI = 205.64–1118.51) respectively (Figs. 4 and 5), indicating a high performance of diagnostic accuracy for CLE in detection of IM among the high-risk group of gastric cancer. The Cochran’s Q and I^2^ for DOR were 2.23 (p = 0.527) and 0, respectively, demonstrating low heterogeneity among the four studies for a per-lesion analysis. Deeks’ funnel plot which was not significantly asymmetrical (Fig. 6) illustrated no significant publication bias in this meta-analysis.

## Discussion

Gastric cancer is still one of the most prevalent and lethal malignant diseases worldwide despite a decrease in its incidence recent decades. Early diagnosis of gastric cancer plays an important role in patients’ prognoses. Gastric intestinal metaplasia (IM) is a relatively frequent precancerous lesion and the progression rate from IM to gastric cancer over 5 years varies from 1.25 to 42 % [19, 20]. Correa et al. reported that incomplete-type IM should be followed by surveillance in order to early diagnosis of dysplasia or early adenocarcinoma [26]. In this context, it may be justified to monitor IM in some patients (such as positive *H. pylori* infection) at high risk of gastric cancer in order to early detection [26, 27]. However, conventional WLE with multiple random biopsies was unable to detect IM effectively because of the significant sampling error [22, 28]. The CLE is a novel endoscopic technique for detection of the gastrointestinal mucosa in vivo with the help of WLE by a microscopy. The new device is integrated into the distal site of the conventional endoscope and can also perform targeted biopsy and virtual histological diagnosis with the help of WLE. Although e-CLE is reported to have a better diagnosis of Barrett’s esophagus and tumors compared with p-CLE [29–31], p-CLE has gained more popularity in detection of various gastrointestinal diseases recently [32]. The p-CLE has a slightly lower resolution and smaller field of view, nevertheless, it is more practical because of greater versatility of its probes and faster frame rate to acquire images [32]. Since microscopic visualization of the gastrointestinal structures could be provided by CLE, the diagnostic criteria of CLE on IM corresponds well with histopathologic criteria. All studies adopted the similar diagnostic criteria proposed by Guo et al. in 2008 [22]. The IM can be identified in CLE if any one of the following features is present in the image field: goblet cells, villiform shape of foveolar epithelium and columnar absorptive cells.

To our knowledge, our meta-analysis was first to summarize the available evidence currently with respect to the diagnostic value of CLE in characterizing of IM. Due to insufficient data, it was impossible to estimate diagnostic value of CLE on a per-patient basis. As mentioned earlier, the summary sensitivity and specificity of CLE were 0.97 (95 % CI = 0.94–0.98) and 0.94 (95 % CI = 0.91–0.97) respectively. The pooled DOR was 479.59 (95 % CI = 205.64–1118.51). These statistical results indicated that CLE had a high level of diagnostic accuracy for IM with the help of WLE. Compared with NBI, which was considered as an extremely useful diagnostic tool for IM [33], CLE had higher sensitivity (96.7 % VS 69 %), similar specificity (94 % VS 91 %) and similar AUC (99 % VS 90 %) on a per-lesion basis [33]. Besides, biopsy examinations required complex and time-consuming procedures. It should be noted that the CLE may not be cost effective compared with the conventional tool [23]. Targeted biopsy by p-CLE was more efficient as less numbers of biopsies are required compared with conventional biopsies. Zhen [23] reported that numbers of biopsies per patient by CLE targeting of biopsies could decrease 68 % compared with standard biopsy protocol. We also searched studies in order to evaluate the diagnostic accuracy of CLE for intraepithelial neoplasia (IN) in this study. Unfortunately, there were few studies. Li [34] reported that CLE had a higher sensitivity (88.9 %), specificity (99.3 %) and accuracy (98.8 %) for identification of gastric cancer or HGIN lesions than WLE diagnosis alone. These data also favored to support that CLE was a promising tool in characterizing of precancerous lesions and early gastric cancer in vivo.

Several precautions should also be considered in this study. First of all, the four studies included in this meta-analysis all came from Asia. There were no similar researches in other regions so far. So researches with high quality data across multiple centers were imperative to evaluate the effectiveness of CLE to diagnose IM. Secondly, not all data could be extracted from the abstracts of studies, another factor of eliminating some studies. At last, only studies published in English were included. The selections might lead to an existing language bias and missing some useful information.

## Conclusion

In summary, CLE is a reliable technique to diagnose IM with high accuracy according to our study. CLE could be considered as a promising endoscopic tool to characterize IM in patients with high risk of gastric cancer.

## Abbreviations

CI: Confidence interval
CLE: Confocal laser endomicroscopy
DOR: Diagnostic odds ratio
FN: False negative
FP: False positive
IM: Intestinal metaplasia
NBI: Narrow-band imaging
NLR: Negative likelihood ratio
PLR: Positive likelihood ratio
QUADAS-2: Quality assessment of diagnostic accuracy studies-2
SROC: Summary receiver operating curve
TN: True negative
TP: True positive
